# Can the relationship between overweight/obesity and sleep quality be explained by affect and behaviour?

**DOI:** 10.1007/s40519-022-01435-1

**Published:** 2022-07-06

**Authors:** S. W. Eid, R. F. Brown, S. K. Maloney, C. L. Birmingham

**Affiliations:** 1 Australian National University, Canberra, Australia; 2 University of Western Australia, Crawley, Australia; 3 University of British Columbia, Vancouver, Australia; 4 Research School of Psychology, ANU College of Health and Medicine, Australian National University, Canberra, ACT 2601 Australia; 5 Unit 3 55 Rosehill Street, Parramatta, NSW 2150 Australia

**Keywords:** Sleep quality, Night-eating, Overweight/obesity, Sleep-disrupting behaviour

## Abstract

**Purpose:**

Sleep impairment is reported to be a consequence of overweight and obesity. However, the weight–sleep relationship can alternately be explained by demographics (e.g. age) and covariates (i.e. mood/affect and behaviour in overweight/obese people; e.g. night-eating). Thus, we examined the weight–sleep quality relationship after controlling for the effects of affect and common behaviour (i.e. night-eating, insufficient exercise, alcohol and electronic device use).

**Methods:**

Online questionnaires asked 161 overweight, obese or normal-weight participants about their sleep quality, night-eating, physical activity, alcohol use, electronic device use and anxiety and depression at T0 (baseline) and T1 (3 months later). Height and weight and waist and hip circumference were objectively measured at T0 and T1, and physical activity was assessed over 24 h (using actigraphy) at T0 and T1. Hierarchical multiple regression analyses evaluated whether the weight measures (i.e. body-mass-index [BMI], waist-to-hip ratio [WHR] and obesity category [overweight/obese vs. normal-weight]) predicted sleep quality and its components at T0 and T1, after controlling demographics (at step 1) and covariates (affective distress and behaviour) at step 2, and entering weight measures at step 3; maximum 8 variables in the analyses.

**Results:**

High BMI predicted several aspects of sleep quality after taking into account co-existing behaviour, affect and demographics: sleep disturbances at T0 and lower sleep efficiency at T1. WHR and obesity category did not predict any aspects of sleep quality. Several co-existing behaviour were related to or predicted sleep quality score and aspects of sleep quality including night-eating, alcohol use and electronic device use and affective symptoms (i.e. anxiety, depression).

**Conclusion:**

Results suggest that a person’s weight may impact on their sleep quality above and beyond the effects of their co-existing behaviour and affect, although their co-existing behaviour and affect may also adversely impact on sleep quality.

**Level of evidence:**

Level III, evidence obtained from well-designed cohort.

## Introduction

Overweight and obesity (e.g. as indexed by high body-mass-index [BMI]) has been shown to be associated with impaired sleep in adults [1, 2] in objective and subjective sleep studies. The finding is interpreted as evidence that impaired sleep is the consequence of a person’s weight [2, 3]. However, as detailed below, the relationship between it and sleep quality may be better explained by the affect (e.g. depression) and behaviour (e.g. night-eating) of overweight people, which may alternately contribute to the sleep impairments.

Clinically, obese patients are reported to have longer awake time, less sleep efficiency (SE; i.e. lower percentage of sleep time during the night), and they are sleepier during the day, relative to normal-weight controls [3]. Similarly, in the community setting, high BMI and overweight/obesity are linked to shorter self-reported sleep duration, but, as it is also linked to high emotional stress, it may not indicate true sleep loss [4]. That is to say, obesity and poor sleep quality often co-exist with emotional stress and *affective distress* (e.g. depression) [5]; for example, anxiety and depression are linked to shorter sleep duration and poor quality sleep [6] and overweight/obese people report higher distress levels than normal-weight people [7]. Thus, affective distress may better explain the documented relationship between weight and sleep.

High BMI has also been shown to be related to excessive daytime sleepiness (EDS) as indexed by objective and subjective sleep tests [5]. In a large obesity study, high BMI, metabolic disturbance (i.e. diabetes, insulin resistance), depression and sleep apnoea all predicted inadequate or disturbed sleep, but so did physical activity levels [8]; suggesting that a sedentary lifestyle and *insufficient activity* can also impair sleep. Similarly, people who fail to meet the activity guidelines (150 min of moderate-intensity, 75 min of vigorous-intensity activity or combination of both) are more likely to report daytime sleepiness than those who do meet them [9] whereas regular activity improves a person’s sleep (e.g. reduced sleep-onset latency [SOL], longer total sleep time) [10]. However, overweight/obese people are more likely to report insufficient physical activity than normal-weight people [11]. Thus, insufficient physical activity may provide a better explanation of the observed weight–sleep relationship.

Excessive *night-eating* may also provide a better explanation of the weight–sleep relationship. It can impair multiple aspects of sleep including subjective difficulty initiating and maintaining sleep [12] and objective sleep impairments (e.g. less SE, long SOL, more sleep arousals) [13]. In addition, once a person’s sleep is impaired, they are more likely to experience increased appetite, lower leptin levels (i.e. adipose tissue hormone suppressing appetite) and higher ghrelin levels (i.e. peptide that stimulates appetite) [14, 15]. Moreover, night-eating is highly correlated with weight (e.g. high BMI, obesity category) [16] and it is likely to contribute to weight gain and overweight/obesity over time [17].

Finally, other behaviour may better explain the weight–sleep relationship. For example, heavy *alcohol use* can interfere with sleep induction and maintenance [18], and it is linked to weight gain and overweight/obesity [19]. Similarly, *electronic device use* can interfere with sleep quality [20] and it is linked to overweight/obesity [21]. Further, demographics may potentially confound the relationship between weight and sleep including *older age* which is linked to difficulty initiating and maintaining sleep [22] as well as higher BMI [23] and less physical activity [24], night-eating [25] and electronic device use [26]) whereas female *gender* is associated with poor sleep quality [27] but less exercise [28] and obesity [29]).

Thus, assorted findings in the literature suggest that the weight–sleep relationship can be better explained by demographics (e.g. gender), affective distress (e.g. depression) and frequent behaviour (e.g. night-eating) in affected individuals. However, it is unclear which of these factors can best account for the weight–sleep relationship as no prior comparison studies have been conducted. Therefore, we examined whether affective distress and frequent behaviour could alternately explain the weight–sleep relationship by examining if the weight–sleep relationship still existed, after controlling for the putative effects of these factors on sleep in the planned analyses.

Specifically, we tested three weight measures (i.e. BMI, waist-to-hip ratio [WHR], obesity category) as predictors of total sleep quality and sleep quality components at baseline (T0) and 3 months later (T1), after controlling for the effects of the aforementioned demographics and covariates (i.e. affective distress and co-existing behaviour). Two measurement time points (T0 and T1) were used to determine whether the observed results were consistent over time; and, a 3-month interval was chosen between T0 and T1 as it permitted sufficient change to occur in the sleep quality components (i.e. dependent variables), which are reported to be labile over short intervals (e.g. 3 months) [30]. However, we did not expect that significant changes will occur in participant’s weight over 3 months [31], although their behaviour and affect may change over 3 months.

Thus, consistent with the prior literature, we expected that the:

1. Measures of weight (BMI, obesity category) at T0 will no longer predict poor total sleep quality at T0 and T1, after controlling for the effects of the aforementioned covariates and demographics; and,
2. Measures of weight (BMI, obesity category) at T0 will no longer predict the components of sleep quality at T0 and T1, after controlling for the effects of the covariates and demographics. Specific hypotheses were not provided for WHR as it has not previously been evaluated in regards to sleep quality. Nonetheless, as it indexes abdominal obesity [32], it permits an examination of whether abdominal obesity is more (or less) strongly related to sleep quality than other measures of weight.

## Methods

### Participants

Participants were recruited via social networking websites (e.g. Facebook, Gumtree), Australian National University (ANU) Psychology Research Participation Scheme, sleep centres (e.g. Canberra Sleep Clinic), local magazines (e.g. Canberra Weekly), and email snowballing. Criteria for study inclusion were: age of 18–65 years, resided in Canberra, Australia, and BMI ≥ 18.5 (BMI = weight (kg)/height (m) squared; World Health Organisation [WHO]) [33], that is, normal-weight, overweight or obese. The study was granted full approval by the ANU Human Research Ethics Committee (protocol #2015/013).

Potential participants (*n* = 165) clicked on the URL embedded in the study advertisement and 162 of them completed the T0 questionnaire (participation rate = 98%), but one did not complete the actigraphy phase of the study, leaving 161 participants. Three months later (T1), most of them (*n* = 155) completed the T1 questionnaire and second actigraphy phase, resulting in an attrition rate of 3.7% (6/161). No remuneration was provided to the participants, but the ANU student participants could receive 3 course credit points for hours of study participation.

### Procedure

Interested individuals clicked on the URL imbedded in the advertisement, read the study information page and indicated their consent to participate. They were then asked to provide their height (in metres) and weight (in kg) to calculate their BMI. If their *self-reported* BMI was < 18.5 they were redirected to the end of the survey and thanked for their time. If their BMI ≥ 18.5, they were asked to complete the T0 questionnaire which asked about demographics, sleep quality, concurrent behaviour and affective distress.

After completing the T0 questionnaire, they were sent an email asking them to organise a time to meet the researcher (SE) face-to-face to fit the physiological monitoring equipment, either in a research room at ANU or in their home. At this meeting, participant’s height, weight, and waist and hip circumferences were *objectively* measured. Height was measured using a Seca 213 Portable Stadiometer and weight was measured using Seca 750 mechanical floor scales. BMI was calculated according to the formula: BMI = weight (kg)/height^2^ (m^2^) [33]. Waist and hip circumference were measured using a 152-cm soft measuring tape wrapped around the narrowest part of the waist and widest part of the hips to calculate WHR: waist circumference (cm)/hip circumference (cm) [34]. *Objectively* measured BMI and WHR were the predictor variables in this study, along with obesity category (overweight/obese vs. normal-weight), using standard WHO categories: normal-weight (BMI: 18.5–24.9), overweight (BMI: 25–29.9), and obese (BMI ≥ 30) [33].

Participants were then fitted with a non-invasive watch-like monitoring device (Actical®) on the wrist, either left or right, according to preference, and using a disposable sterile wristband, to objectively monitor their physical activity levels over 24 h. The Actical® system’s menu was used to configure the devices for each participant, by inputting their study ID number, age, gender, height and weight. Participants wore the devices continuously for 24 h, after which time, they returned them to the researcher.

Three months later, participants were sent an email with a URL link to the T1 questionnaire which asked them the same questions as the T0 questionnaire. After completing the questionnaire, they were again asked to meet the researcher face-to-face to have their height, weight, and waist and hip circumference objectively measured and the Actical® device fitted. They wore the device for 24 h and then returned it to the researcher. No participants removed their device during the two 24-h monitoring periods.

### Apparatus

*Physical activity* (total number of steps) was assessed over 24 h at T0 and T1 using the Actical® accelerometers (Phillips, Respironics), which tolerate normal daily activities such as showering. The devices recorded activity using a 1-min epoch length. Data held on the devices were downloaded using ActiReader® communications interface software via a wireless link. The devices have been used to monitor physiological [35, 36] and behavioural parameters [37] in humans and they show moderate convergent validity with other objective measures of physical activity [38].

### Materials

*Sleep quality* was assessed using the Pittsburgh Sleep Quality Index (PSQI; 19 items) which assesses total sleep quality, subjective sleep quality (SSQ), sleep-onset latency (SOL), sleep duration, habitual sleep efficiency (SE), sleep disturbances (SD), sleep medication use (SM) and daytime dysfunction (DD). Participants were asked to rate their agreement with each item using 4-point scales ranging from 0 (*not in the past month*) to 3 (*three or four times a month*), with high scores indicating poorer sleep. PSQI is a valid measure of sleep quality in clinical and non-clinical samples: it distinguishes between good vs. poor sleepers and it has moderate convergent validity with objective sleep measures [39] and information collated from sleep diaries [40]. PSQI is reported to have high internal consistency with Cronbach’s α (CA) of 0.80 [41]. In this study, PSQI total score had adequate internal consistency at T1 with CA of 0.72 and SD (9 items) had a CA of 0.63. Internal consistency was not calculated for the other PSQI subscales as they were comprised of only one or two items.

*Physical activity* was assessed using the Rapid Assessment of Physical Activity (RAPA; 9 items) [42] which asks about exercise intensity, using yes/no responses. Participants were provided with a definition of physical activity and examples of light, moderate and vigorous activity to assist in rating their current activity on two scales: physical activity and strength/flexibility. RAPA has adequate test–retest reliability (*r* = 0.65) [43] and is a valid measure of activity that distinguishes between people who do/do not undertake moderate exercise [44]. In this study, RAPA had adequate test–retest reliability (*r* = 0.72) over 3 months. Using the RAPA, participants were considered to be sufficiently active [45] if they had spent ≥ 150-min engaged in moderate-intensity activity or 75-min vigorous-intensity activity/week.

Physical activity was also assessed using the Global Physical Activity Questionnaire-Version 2 (GPAQv2) [46, 47] that collects activity information across three domains: occupational, transportation and leisure-time activity. GPAQ asks if a person has engaged in moderate or vigorous-intensity exercise in each activity domain. If they answered yes, they were asked how many days they engaged in the activities in a typical week and how long they spent doing the activities on a typical day. GPAQ data was cleaned and screened using GPAQ guidelines [48] and sub-scores were calculated for each activity domain based on the average number of days, hours and minutes spent doing the activity each week. A measure of total physical activity (GPAQ score) taking into account the intensity of the activities (by way of metabolic equivalents [METs]) was calculated. GPAQ score has adequate test–retest reliability (*r* = 0.67) [49] and it is a valid measure of physical activity [47]. In this study, GPAQ score had adequate test–retest reliability (*r* = 0.64) over 3 months and it was more highly correlated with the actigraphy-derived activity data suggesting that it more closely indexed whether participants met the CDC requirements for optimal physical activity [50]. Although RAPA and GPAQ scores were moderately correlated (*r* = 0.32–0.47), RAPA scores were poorly correlated with the objective activity measures (see supplementary file).

*Night-eating* was assessed using the Night-Eating Questionnaire (NEQ, 14 items) that detects the presence of night-eating syndrome (NES) in adults across four subscales: morning anorexia, evening hyperphagia, nocturnal ingestions and mood/sleep problems. It also detects night-eating in people without NES (e.g. university students) [51] especially in high stress situations [52]. Night-eating severity was assessed using 5-point scales ranging from 0 (*not at all*) to 4 (*very often*), with high scores indicating more night-eating. NEQ score ≥ 25 indicates possible NES. NEQ score has adequate internal consistency with a CA of 0.7 and good convergent validity [53]. In this study, internal consistency of total NEQ score was adequate with a CA of 0.66 at T1. Criteria for NES include: consuming ≥ 50% of daily energy intake after the evening meal, eating after waking from sleep and morning anorexia [54].

*Other behaviour* (that can disrupt sleep) was examined including alcohol use and electronic device use (including mobile phone and TV use). Participants were asked if they had consumed alcohol or engaged in the activity (yes/no), and if so, the amount (1–5 to > 20 drinks), frequency (every day, 3–4 times per week, twice a week, once a week, once a fortnight, once a month, < once a month, never) and time of day (morning, afternoon, evening, late night) of the use/behaviour. Participants listed all the electronic devices they had used in a typical day along with the number of hours each day and the time/s of day of the use.

*Affective distress* was assessed using the Depression Anxiety Stress Scales-21 (DASS-21; 21 items) that assesses the presence and severity of depression and anxiety symptoms. Participants were asked to rate each item using 4-point scales ranging from 0 (*did not apply to me at all*) to 3 (*applied to me very much/most of the time*), with high scores reflecting more distress (e.g. severe depression (DASS-D > 21) and severe anxiety (DASS-A > 15). The scale has high internal consistency with CAs of 0.84 for anxiety and 0.91 for depression [55] in non-clinical [56] and clinical samples [57]. In this study, its internal consistency was high with CAs of 0.88 for depression and 0.81 for anxiety.

### Statistical analyses

An a priori power analysis using G*power (version 3.0.10) estimated that 139 people were required to detect a medium effect size (ƒ^2^ = 0.15) with alpha set at 0.05, power of 0.8 and using up to 10 predictors in the analyses. Thus, 161 participants were deemed sufficient to conduct the cross-sectional analyses and 155 participants were sufficient for the longitudinal analyses.

Routine statistical analyses were performed using the statistical analysis program SPSS (Version 24). Analysis of Variance (ANOVA) and Chi-square tests evaluated whether the study variables differed between T0 and T1. Hierarchical multiple regression analyses examined the predictors of total sleep quality and PSQI sub-scores (SSQ, SOL, sleep duration, SE, SD, SM, DD) at T0 and T1, after controlling for the effects of demographics (i.e. age, gender) at step 1 of the analysis. Covariates (i.e. behaviour and affective distress) were entered at step 2 and the weight predictor variables (i.e. BMI, WHR, obesity category) were entered at step 3 of the analyses. As a result of the large number of variables in the study, only those that were significantly correlated with the sleep quality variables were included in the analyses, with significance set at *p* < 0.05.

## Results

Means, standard deviations and correlations between the study variables are presented in Tables 1a–i, 2 (see supplementary file for Tables 1a–i).

**Table 1:** Sample characteristics at baseline T0 (*N* = 161)

Measure	Number	%
Sleep quality (PSQI score ≥ 5)	134	83
SOL (minutes) > 30 min	87	54
Sleep duration (hours) < 7 h	29	18
Sleep efficacy (%) < 85%;	68	42
Sleep disturbance (% of sleep)	157	98
Daytime dysfunction (moderate)	134	83
Sleep medication use (%)	28	17
Obese	36	22
Overweight	35	22
Normal weight	90	56
Sufficiently active (RAPA)	47	29
Optimal PA (GPAQ)	113	80
NEQ score > 25	22	14
Severe depression	30	19
Severe anxiety	52	32

**Table 2:** Means and standard deviations of key study variables at T0 and T1 (N = 161)

	T0 (*N* = 161)	T1 (*N* = 155)	T0 vs T1
	M (SD)	M(SD)	
Subjective sleep quality^	1.53 (.73)	1.18 (0.68)	*F*(1, 154) = 33.23, *p* < 0.001**
Sleep latency (min)	1.67 (1.02)	1.42 (0.98)	*F*(1, 154) = 9.66, *p* = 0.002**
Sleep duration (hrs)^	0.96 (0.76)	0.83 (0.75)	*F*(1, 154) = 4.56, *p* = 0.03*
Habitual sleep efficacy^	0.66 (0.91)	0.73 (1.02)	*F*(1, 154) = 0.72, *p* = 0.40
Sleep disturbances (number)	1.43 (0.54)	1.21 (0.48)	*F*(1, 154) = 29.80, *p* < 0.001**
Sleep medication use	0.30 (0.76)	0.33 (0.77)	*F*(1, 154) = 0.05, *p* = 0.82
Daytime dysfunction	1.45 (0.77)	1.04 (0.73)	*F*(1, 154) = 33.74, *p* < 0.001**
PSQI^	7.99 (3.42)	6.63 (3.28)	*F*(1, 154) = 32.64, *p* < 0.001**
NEQ	17.30 (6.67)	15.84 (6.52)	*F*(1, 154) = 7.26, *p* = 0.01*
Depression	13.18 (9.57)	11.69 (10.49)	*F*(1, 154) = 5.17, *p* = 0.02*
Anxiety	11.79 (8.92)	9.90 (8.32)	*F*(1, 154) = 9.87, *p* = 0.002**
RAPA: activity level	4.37 (1.87)	4.44 (1.92)	*F*(1, 154) = .08, *p* = 0.78
RAPA: strength/flexibility	1.05 (1.13)	1.12 (1.22)	*F*(1, 154) = .59, *p* = 0.44
GPAQ total	2407.60 (3274.67)	2159.68 (2970.70)	*F*(1, 154) = 1.32, *p* = 0.25
Alcohol intake (# drinks)	3.48 (2.69)	0.59 (0.99)	*F*(1, 154) = 224.9, *p* < 0.001**
TV time (h)	1.57 (0.82)	2.67 (0.92)	*F*(1, 154) = 393, *p* = 0.001**

^ Variable inversely coded. *p < 0.05. **p < 0.005

### Sample description

Of the 161 participants, 67 (42%) were male and 94 (58%) were female and the mean age was 26.8-years (range: 18–65 years, SD = 9.45). Repeated-measures ANOVA showed their age did not vary significantly between T0 and T1 (*M* = 26.8 vs. 26.9 years, SD = 9.5 vs. 9.6, *F*_33, 121_ = 0.98, *p* = 0.52). Chi-square tests showed that they did not differ in terms of gender (χ^2^_1_ = 0.49, *p* = 0.48), marital status (χ^2^_5_ = 4.42, *p* = 0.49), education (χ^2^_4_ = 3.05, *p* = 0.55) or employment (χ^2^_6_ = 2.57, *p* = 0.86) between T0 and T1. Nearly two-thirds of them were single (62%, *N* = 100), 24% (*N* = 38) were married and the rest were divorced/separated (*N* = 5), lived with someone (*N* = 12) or did not want to say (*N* = 6). More than one-half had finished an undergraduate (29%, *N* = 47) or postgraduate (24%, *N* = 39) degree and the rest had finished a diploma (*N* = 9) or Year 12 (39%, *N* = 63) or Year 10 or below at high school (*N* = 3). More than one-half of them were students (60%, *N* = 96), 20% worked full-time (*N* = 32) and the rest worked part-time/casual (16%, *N* = 26), completed home duties (*N* = 3), were unemployed (*N* = 1) or permanently unable to work or ill (*N* = 2).

At baseline (T0), in terms of sleep, 83% of them had poor sleep quality (PSQI score ≥ 5; PSQI cutoff score) [39]. One-half of them took longer than 30 min to fall asleep (SOL), nearly 20% had sleep duration < 7 h, 42% had low sleep efficiency (SE < 85%), 98% reported sleep disturbances (SD), 83% had moderate daytime dysfunction (DD), and 17% used sleep medications (SM). In terms of weight, over one-half of them was normal weight, 22% were overweight, and 22% were obese at T0, using WHO weight classifications [33]; see Table 1.

In terms of behaviour and affect, few (14%) reported substantial night-eating (NEQ score > 25) [58] but many of them (70%) were sufficiently active using GPAQ [50] although fewer (29%) were sufficiently active using RAPA cut-off scores [45]; but as the latter figure is closest to the actigraphy-derived data, GPAQ score most likely accurately reflected activity levels. Finally, some participants reported severe depression (19%) and/or anxiety (32%) symptoms relative to DASS-21 cutoff scores [55]. Thus, relative to Australian adult prevalence figures or normed values, fewer of them was overweight/obese [59], physically inactive [45] and clinically significant night-eaters [25] than was expected, but more of them were affectively distressed [60]; see Table 2.

Three months later (baseline [T0] to [T1]), participant’s sleep quality slightly improved (SSQ, SOL, sleep duration, SD, DD) as did their night-eating, anxiety and depression, and their alcohol intake decreased and TV viewing increased; see Table 2. Such small changes are not likely due to demographic changes over time as only six people dropped out of the study before T1. It is likely that the changes were due to normal fluctuations in symptoms and behaviour that are linked to seasonal changes [61, 62] or changing life circumstances (e.g. life-event stress) [63, 64].

Described below are a series of hierarchical multiple regression analyses evaluating whether the weight measures still predicted total sleep quality score and the seven sleep quality components, at T0 and T1, after controlling for the effects of the aforementioned affective and behavioural measures.

### Predictors of total PSQI score

Cross-sectional predictors of sleep quality (PSQI score) were examined at T0. Covariates (i.e. depression, anxiety, alcohol intake, work-physical activity, and night-eating) were entered at step 1 of the analysis but as no demographics or weight variables were correlated with it they were not entered into the analysis. In the analysis, anxiety and depression levels, night-eating and alcohol intake were associated with high PSQI score, but not the weight measures; see Table 3.

**Table 3:** Predictors of PSQI at T0 (*N* = 161) and T1 (*N* = 155)

Variable	*B*	SE	*B*	*t*	*p*	R2
T0 Step 1						
Depression	0.09	0.029	0.252	3.137	0.002**	
Anxiety	0.084	0.032	0.219	2.629	0.009*	0.398**
Alcohol intake	0.186	0.08	0.146	2.325	0.021*	
Work PA	0.0	0.0	0.104	1.608	0.11	
NEQ	0.144	0.034	0.281	4.214	0.000**	
T1 Step 1						
PSQI T0	0.591	0.06	0.624	9.869	0.000**	0.389**
T1 Step 2						
PSQI T0	0.542	0.076	0.572	7.098	0.000**	0.401**
Depression	0.006	0.029	0.018	0.215	0.83	
Anxiety	− 0.006	0.031	− 0.016	− 0.187	0.852	
Work PA	0.0	0.0	0.103	1.564	0.12	
NEQ	0.032	0.037	0.063	0.86	0.391	

*p < 0.05. **p < 0.005

Longitudinal predictors of sleep quality (PSQI score) were examined at T1. PSQI T0 and age were entered at step 1 and covariates (i.e. depression, anxiety, work-physical activity, and night-eating) were entered at step 2 of the analysis. None of the weight measures was correlated with it so they were not entered into the analysis. In the analysis, none of the variables predicted PSQI score at T1, after controlling for the effects of T0 PSQI score and age; see Table 3.

### Predictors of subjective sleep quality

Cross-sectional predictors of SSQ were examined at T0. Demographics (i.e. age) were entered at step 1, covariates (i.e. depression, anxiety, work-physical activity, alcohol intake, and night-eating) were entered at step 2 and the weight variables (i.e. WHR) were entered at step 3 of the analysis. After controlling for the effects of the variables, only depression levels, alcohol intake and night-eating were related to poor SSQ; see Table 4.

**Table 4:** Predictors of subjective sleep quality and sleep-onset latency at T0 (*N* = 161) and T1 (*N* = 155)

Variable	*B*	SE	*B*	*t*	*p*	R2
Subjective sleep quality						
T0 Step 1						
Age	− 0.016	0.006	− 0.208	− 2.683	0.008*	0.043*
T0 Step 2						
Age	− 0.011	0.006	− 0.143	− 1.996	0.048*	0.301**
Depression	0.023	0.007	0.298	3.432	0.001**	
Anxiety	0.006	0.008	0.068	0.744	0.458	
Work PA	0.0	0.0	0.125	1.756	0.081	
Alcohol intake	0.042	0.019	0.155	2.203	0.029*	
NEQ	0.022	0.008	0.199	2.759	0.007*	
T0 Step 3						
Age	− 0.01	0.006	− 0.123	− 1.576	0.117	0.303**
Depression	0.023	0.007	0.297	3.41	0.001**	
Anxiety	0.006	0.008	0.07	0.764	0.446	
Work PA	0.0	0.0	0.121	1.693	0.093	
Alcohol intake	0.042	0.019	0.154	2.188	0.03*	
NEQ	0.021	0.008	0.192	2.625	0.01*	
WHR	− 0.457	0.692	− 0.049	− 0.66	0.51	
T1 Step 1						
Subjective sleep quality 0	0.416	0.067	0.448	6.196	0.000**	0.201**
T1 Step 2						
Subjective sleep quality 0	0.332	0.077	0.358	4.306	0.000**	0.265**
Depression	0.008	0.007	0.115	1.183	0.239	
Anxiety	− 0.006	0.007	− 0.077	− 0.812	0.418	
Alcohol intake	0.04	0.019	0.156	2.137	0.034*	
Steps	0.0	0.0	0.129	1.778	0.077	
NEQ	0.012	0.008	0.111	1.421	0.157	
Sleep latency						
T0 Step 1						
Age	− 0.017	0.008	− 0.159	− 2.034	0.044*	0.025*
T0 Step 2						
Age	− 0.008	0.008	− 0.075	− 0.989	0.324	0.212**
Depression	0.009	0.01	0.084	0.907	0.366	
Anxiety	0.014	0.011	0.126	1.314	0.191	
Alcohol intake	0.069	0.038	0.183	1.817	0.071	
Alcohol frequency	− 0.022	0.112	− 0.019	− 0.194	0.846	
NEQ	0.045	0.012	0.296	3.823	0.000**	
T1 Step 1						
Sleep-onset latency T0	0.594	0.061	0.622	9.816	0.000**	0.404**
Age	− 0.007	0.006	− 0.068	− 1.072	0.285	
T1 Step 2						
Sleep-onset latency T0	0.58	0.067	0.607	8.689	0.000**	0.425**
Age	− 0.007	0.007	− 0.07	− 1.059	0.291	
Depression	0.003	0.008	0.027	0.337	0.737	
Anxiety	0.005	0.009	0.045	0.531	0.596	
Alcohol intake	− 0.028	0.032	− 0.076	− 0.867	0.387	
Alcohol frequency	0.188	0.093	0.171	2.012	0.046*	
NEQ	− 0.007	0.011	− 0.046	− 0.647	0.519	

*p < 0.05. **p < 0.005

Longitudinal predictors of SSQ were examined at T1. T0 SSQ was entered at step 1, covariates (i.e. depression, anxiety, alcohol intake, # steps, night-eating) were entered at step 2. No weight variables were correlated with SSQ so they were not entered in the analysis. After controlling for the effects of T0 SSQ, only alcohol intake predicted SSQ at T1; see Table 4.

### Predictors of sleep-onset latency

Cross-sectional predictors of SOL were examined at T0. Demographics (i.e. age) were entered at step 1, covariates (i.e. depression, anxiety, alcohol intake, alcohol frequency, and night-eating) were entered at step 2 but as no weight variables were correlated with it they were not entered into the analysis. After controlling for the effects of the variables, only night-eating was related to longer SOL; see Table 4.

Longitudinal predictors of SOL were examined at T1. T0 SOL and age were entered at step 1 and covariates (i.e. depression, anxiety, alcohol intake, alcohol frequency, and night-eating) were entered at step 2. No weight variables were correlated with it so they were not entered into the analysis. After controlling for the effects of the variables, only frequency of alcohol intake predicted SOL at T1; see Table 4.

### Predictors of sleep duration

Cross-sectional predictors of sleep duration were examined at T0. No demographics or weight measures were correlated with it so they were not entered into the analysis and as only anxiety was correlated with it, only anxiety was entered at step 1 of the analysis and only it was related to shorter sleep duration at T0; see Table 5

**Table 5:** Predictors of sleep duration, sleep efficacy, and sleep disturbances at T0 (*N* = 161) and T1 (*N* = 155)

Variable	*B*	SE	*B*	*t*	*p*	R2
Sleep duration						
T0 Step 1						0.027**
Anxiety	0.014	0.007	0.165	2.115	0.04*	
T1 Step 1						0.088**
Sleep duration T0	0.228	0.108	00.165	2.11	0.037*	
Age	0.014	0.006	00.179	2.293	0.023*	
Gender	0.22	0.119	00.145	1.854	0.066	
T1 Step 2						0.101**
Sleep duration T0	0.16	0.117	0.116	1.369	0.173	
Age	0.015	0.006	0.187	2.401	0.018*	
Gender	0.209	0.119	0.137	1.757	0.081	
NEQ	0.015	0.01	0.123	1.456	0.148	
Sleep efficacy						
T0 Step 1						0.151**
Depression	0.006	0.009	0.066	0.697	0.487	
Anxiety	0.019	0.01	0.19	1.931	0.055	
Work PA	0.0	0.0	0.107	1.404	0.162	
NEQ	0.027	0.011	0.195	2.48	0.014*	
T1 Step 1						0.219**
Sleep efficacy T0	0.44	0.081	0.391	5.408	0.001**	
Gender	0.438	0.149	0.212	2.937	0.004**	
T1 Step 2						0.261**
Sleep efficacy T0	0.42	0.081	0.374	5.211	0.001**	
Gender	0.419	0.146	0.203	2.865	0.005**	
Work PA	0.00	0.00	0.064	0.884	0.378	
Device use	− 0.219	0.084	− 0.186	− 2.606	0.01*	
T1 Step 3						0.302**
Sleep efficacy T0	0.409	0.079	0.364	5.194	0.001**	
Gender	0.405	0.143	0.196	2.84	0.005**	
Work PA	0.00	0.00	0.071	1.007	0.316	
Device use	− 0.17	0.084	− 0.144	− 2.025	0.045*	
BMI	0.031	0.01	0.207	2.956	0.004**	
Sleep disturbances						
T0 Step 1						0.238**
Depression	0.016	0.005	0.285	3.179	0.002**	
Anxiety	0.005	0.006	0.086	0.941	0.348	
NEQ	0.021	0.006	0.256	3.447	0.001**	
T0 Step 2						0.264**
Depression	0.016	0.005	0.277	3.138	0.002**	
Anxiety	0.005	0.006	0.076	0.838	0.403	
NEQ	0.022	0.006	0.266	3.626	0.000**	
BMI	0.013	0.006	0.161	2.33	0.021*	
T1 Step 1						0.281**
Sleep disturbances T0	0.466	0.06	0.53	7.737	0.001**	
T1 Step 2						0.292**
Sleep disturbances T0	0.436	0.071	0.497	6.187	0.000**	
Depression	0.001	0.005	0.019	0.208	0.836	
Anxiety	0.001	0.005	0.017	0.185	0.853	
Work PA	0.0	0.0	0.094	1.314	0.191	
NEQ	0.001	0.006	0.015	0.192	0.848	

*p < 0.05. **p < 0.005

Longitudinal predictors of sleep duration were examined at T1. T0 sleep duration, age, and gender were entered at step 1 and night-eating was entered at step 2 but no weight variables predicted sleep duration at T1, so they were not entered into the analysis. Only older age was found to predict shorter sleep duration at T1 at step 2; see Table 5.

### Predictors of habitual sleep efficacy

Cross-sectional predictors of SE were examined at T0. No demographics or weight measures were correlated with sleep duration so they were not entered into the analysis. Covariates (i.e. depression, anxiety, work-physical activity, and night-eating) were, therefore, entered at step 1 of the analysis. After controlling for the effects of the variables, only night-eating was related to less SE at T0; see Table 5.

Longitudinal predictors of SE were examined at T1. T0 SE and gender were entered at step 1, covariates (i.e. work-physical activity, electronic device use) were entered at step 2, and the weight variables (i.e. BMI) were entered at step 3 of the analysis. After controlling for the effects of the variables, female gender, electronic device use and high BMI predicted lower SE at T1; see Table 5.

### Predictors of sleep disturbances

Cross-sectional predictors of SD were examined at T0. No demographics were correlated with it so they were not entered into the analysis. Covariates (i.e. depression, anxiety, and night-eating) were entered at step 1 and the weight variables (i.e. BMI) were entered at step 2 of the analysis. After controlling for the effects of the variables, higher depression levels, night-eating and high BMI were related to more SD at T0; see Table 5.

Longitudinal predictors of SD were examined at T1. T0 SD was entered at step 1 and covariates (i.e. depression, anxiety, work-physical activity, and night-eating) were entered at step 2 but as no demographics or weight measures were correlated with it at T1 they were not entered into the analysis. After controlling for the effects of the variables, none of the variables predicted SD at T1; see Table 5.

### Predictors of sleep medication use

Cross-sectional predictors of SM were examined at T0. No demographics were correlated with SD at T1 so they were not entered into the analysis. Covariates (i.e. depression, anxiety, work-physical activity, electronic device use, and night-eating) were entered at step 1 and the weight measures (i.e. obesity category) were entered at step 2 of the analysis. After controlling for the effects of the variables, only electronic device use was related to less SM use; see Table 6.

**Table 6:** Predictors of sleep medication use and daytime dysfunction at T0 (*N* = 161) and T1 (*N* = 155)

Variable	B	SE	*B*	*t*	*p*	R2
Sleep medication use						
T0 Step 1						0.145**
Depression	0.007	0.008	0.092	0.965	0.336	
Anxiety	0.012	0.008	0.142	1.432	0.154	
Work PA	0.0	0.0	0.141	1.804	0.073	
Device use	− 0.161	0.066	− 0.185	− 2.441	0.016*	
NEQ	0.012	0.009	0.107	1.351	0.179	
T0 Step 2						
Depression	0.007	0.008	0.091	0.954	0.341	0.160**
Anxiety	0.012	0.008	0.139	1.412	0.16	
Work PA	0.0	0.0	0.138	1.775	0.078	
Device use	− 0.136	0.068	− 0.155	− 2.006	0.047*	
NEQ	0.012	0.009	0.104	1.321	0.188	
Obesity category	0.193	0.117	0.126	1.648	0.101	
T1 Step 1						
Use of sleeping medication T0	0.581	0.065	0.579	8.888	0.001**	0.359**
Age	0.01	0.005	0.121	1.859	0.065	
T1 Step 2						
Use of sleeping medication T0	0.547	0.069	0.545	7.896	0.001**	0.370**
Age	0.01	0.006	0.12	1.663	0.098	
Depression	0.006	0.005	0.08	1.176	0.242	
Work PA	0.00	0.00	0.064	0.937	0.351	
Device use	− 0.008	0.066	− 0.009	− 0.128	0.898	
T1 Step 3						0.371**
Use of sleeping medication T0	0.543	0.07	0.541	7.725	0.001**	
Age	0.009	0.006	0.107	1.381	0.169	
Depression	0.006	0.006	0.079	1.162	0.247	
Work PA	0.00	0.00	0.064	0.942	0.348	
Device use	− 0.007	0.066	− 0.008	− 0.105	0.916	
Obesity category	0.049	0.114	0.032	0.433	0.666	
Daytime dysfunction						
T0 Step 1						0.059**
Age	− 0.02	0.006	− 0.244	− 3.166	0.002**	
T0 Step 2						
Age	− 0.012	0.006	− 0.153	− 2.193	0.03*	0.309**
Depression	0.024	0.007	0.303	3.528	0.001**	
Anxiety	0.012	0.008	0.144	1.613	0.109	
Alcohol intake	0.046	0.02	0.164	2.376	0.019*	
NEQ	0.018	0.008	0.155	2.179	0.031*	
T0 Step 3						
Age	− 0.008	0.006	− 0.101	− 1.29	0.199	0.325**
Depression	0.024	0.007	0.3	3.502	0.001**	
Anxiety	0.014	0.008	0.161	1.808	0.073	
Alcohol intake	0.043	0.02	0.151	2.192	0.03*	
NEQ	0.019	0.008	0.163	2.262	0.025*	
Obesity category	− 0.233	0.122	− 0.15	− 1.905	0.059	
WHR	0.265	0.755	0.028	0.351	0.726	
T1 Step 1						
Daytime dysfunction T0	0.313	0.076	0.318	4.146	0.000**	0.101**
Daytime dysfunction T0	0.161	0.086	0.163	1.881	0.062	0.209**
Depression	0.01	0.008	0.134	1.31	0.192	
Anxiety	0.012	0.008	0.155	1.514	0.132	
Physical activity (RAPA)	0.022	0.038	0.055	0.577	0.565	
Steps	0.0	0.0	0.124	1.548	0.124	
Work PA	0.0	0.0	0.057	0.714	0.476	
Leisure-time PA	0.0	0.0	0.055	0.593	0.554	
NEQ	0.005	0.009	0.041	0.497	0.62	

*p < 0.05. **p < 0.005

Longitudinal predictors of SM were examined at T1. SM T0 and age were entered at step 1, covariates (i.e. depression, work-physical activity, and device use) were entered at step 2, and the weight variables (i.e. obesity category) were entered at step 3 of the analysis. After controlling for the effects of the variables, none of the variables predicted SM use; see Table 6.

### Predictors of daytime dysfunction

Cross-sectional predictors of DD were examined at T0. Age was entered at step 1, covariates (i.e. depression, anxiety, alcohol intake, and night-eating) were entered at step 2, and the weight variables (i.e. obesity category and WHR) were entered at step 3 of the analysis. After controlling for the effects of the variables, only depression, alcohol intake and night-eating were related to more DD at T0; see Table 6.

Longitudinal predictors of DD were examined at T1. T0 DD was entered at step 1 and covariates (i.e. depression, anxiety, physical activity-RAPA, # steps, work-physical activity, leisure time-physical activity, and night-eating) were entered at step 2 but as no demographics or weight measures were correlated with it so they were not entered into the analysis. After controlling for the effects of the variables, no variables predicted DD at T1; see Table 6.

## Discussion

Impaired sleep is reported to be a consequence of overweight/obesity in adults [1, 2], but their behaviour and affective distress (e.g. anxiety, depression) may provide a better explanation of the weight–sleep relationship. That is, certain behaviour (e.g. night-eating, insufficient physical activity, heavy alcohol and electronic device use) are practised more often by overweight/obese people than normal-weight people [11, 16, 19, 21], and the behaviour (and affect) are independent risk factors for impaired sleep [13, 63, 65–67]. Thus, an overweight person’s behaviour and affect may adversely impact on their sleep quality more so than their weight. We, therefore, evaluated if weight (i.e. BMI, WHR, obesity category) still predicted aspects of sleep quality after controlling for the effects of demographics, co-existing behaviour and affective symptoms (i.e. covariates). Hypothesis 1 evaluated weight predictors of total sleep quality score and hypothesis 2 examined the predictors of the seven sleep quality components.

### *Hypothesis 1*

examined the weight measures as predictors of total sleep quality score. No weight measures predicted it at T0 or T1 after controlling for the effects of co-existing behaviour, affect and demographics, supporting the null hypothesis. Results indicated that the weight measures failed to predict sleep quality score at baseline and 3 months later, after taking into account the participant’s co-existing behaviour, affect and demographics.

### *Hypothesis 2*

examined the weight measures as predictors of the sleep quality components (SSQ, SOL, sleep duration, SE, SD, DD and SM) at T0 and T1, after controlling for the effects of co-existing behaviour, affect and demographics. Contrary to expectation, some weight measures still predicted sleep quality after taking into account multiple co-existing behaviour, affect and demographics: higher BMI still predicted more sleep disturbances (SD) at T0 and lower sleep efficiency (SE) at T1, but WHR and obesity category did not predict aspects of sleep quality. Results suggest that a person’s weight may impact on their sleep quality above and beyond the effects of their co-existing behaviour and affect. However, abdominal obesity (as indexed by WHR) [32] was unrelated to sleep quality, suggesting that it was not a specific risk factor for impaired sleep in overweight/obese individuals.

Finally, regarding the covariates, at T0, night-eating was related to sleep quality score and five out of seven of the sleep quality components (SSQ, SOL, SE, SD, and DD), whereas other covariates were linked to fewer deficits: depression was related to sleep quality score, SSQ, SD and DD at T0; anxiety was related to sleep quality score and sleep duration at T0; alcohol use (or frequency) was related to sleep quality score, SSQ and DD at T0 and SSQ and SOL at T1; and, electronic device use predicted SM use at T0 and SE at T1. Results suggest that participant’s co-existing behaviour (i.e. night-eating, electronic device and alcohol use) and their affect (i.e. anxiety/depression) may have adversely impacted on their sleep quality, aside from the putative effects of body weight on sleep quality.

Taken together, the study results suggest that a person’s weight may still impact on their sleep quality even after taking into account the effects of co-existing behaviour and affect, but their affect and co-existing behaviour may also have adversely impacted on their sleep quality. Results are consistent with prior research showing that impaired sleep (e.g. short sleep duration) is related to measures of weight (e.g. high BMI, obesity category), [14, 68], behaviour including night-eating (i.e. long SOL, less SE, sleep maintenance) [12, 13, 51], excessive alcohol use [64] and electronic device use [69] and affective symptoms such as anxiety [70] and depression [71, 72], but we did not find any relationship between sleep and physical activity as have other studies [73]. However, the results were different to those obtained using the *same sample* and weight measures in an objective sleep study [74], in which weight (and night-eating) failed to predict *objective* sleep. Instead, it was predicted by a combination of other behaviour (i.e. alcohol use, watching TV, and physical inactivity) and affect (i.e. depression). Thus, taken together, the results suggest that a different profile of behaviour may interfere with objective vs. subjective sleep; for example, night-eating, weight and affect may interfere with subjective sleep whereas affect and other behaviour (e.g. watching TV) may interfere with objective sleep; aside from affirming that subjective sleep quality does not provide reliable information about objective sleep [75].

*Clinically*, the results suggest that a person’s weight may still impact on their sleep quality (e.g. less sleep efficiency and more sleep disturbances) above and beyond the effects of their co-existing behaviour and affect. However, many participants were distressed and they engaged in multiple potentially sleep-disruptive behaviour [6], in particular, the people who reported more night-eating were most likely to report multiple aspects of poor sleep quality as well as being more likely to be overweight/obese [16].

Nonetheless, the study results need to be interpreted in light of several *study limitations*. First, the sample was relatively small but an a priori power analysis showed that it was sufficient to detect expected medium effect sizes. Second, sleep was assessed using a self-report scale rather than objective sleep tests, which can result in over- or under-reporting [76], but PSQI has good psychometric properties [39], and we examined objective sleep in this sample in another study [74]. Third, the NEQ had barely adequate internal consistency in this study suggesting the results for night-eating should be interpreted with caution. Further, NEQ score includes a sub-score for mood/sleep problems [53] potentially resulting in the double counting of sleep deficits in the participants (i.e. PSQI and NEQ), which in turn, may have exaggerated the strength of the observed relationship. Fourth, alcohol and electronic device use were examined using an un-validated instrument, but they were only covariates (not predictors) in the planned analyses. Finally, the use of an online survey platforms to deliver questionnaires can attract younger and better educated adults as participants [77] which may have reduced the generalisability of the results to older and less educated people.

## Conclusions

Measures of weight (i.e. BMI, WHR, and obesity category) were examined as predictors of sleep quality at two time-points 3 months apart (T0 and T1) in a community sample of normal-weight and overweight or obese adults, after controlling for the effects of co-existing behaviour, affect (i.e. *covariates*; e.g. night-eating, insufficient physical activity, alcohol use, electronic device use, and anxiety/depression symptoms) and demographics, all of which co-exist with overweight/obesity and independently predict sleep outcomes. Results showed that high BMI predicted several aspects of sleep quality: sleep disturbances at T0 and low sleep efficiency at T1, after taking into account co-existing behaviour, affect and demographics. WHR and obesity category failed to predict aspects of sleep quality. Several co-existing behaviour were associated with or predicted sleep quality score and aspects of sleep quality including night-eating, alcohol use, electronic device use and affective symptoms (i.e. anxiety, depression). Results suggest that a person’s weight may still impact on their sleep quality above and beyond the effects of co-existing behaviour and affect, although co-existing behaviour and affect may also adversely impact on their sleep quality. Clinically, the results suggest that multiple potential risk factors for impaired sleep should be examined when investigating the sleep disturbance in overweight clients as they may provide a better explanation of the impaired sleep than their weight.

1. What is already known on this subject?

Impaired sleep is reported to be a consequence of overweight/obesity. However, the behaviour that overweight people engage in (e.g. night-eating, insufficient physical activity) and affective distress (e.g. anxiety, depression) are independent risk factors for impaired sleep which might alternately explain the observed weight–sleep quality relationship.

Thus, it is unclear whether body weight still explains the observation of impaired sleep in overweight/obese people after taking into account their co-existing behaviour and affect.

2. What does this study add?

Relationships between weight measures (i.e. BMI, WHR, and obesity category) and the components of sleep quality were examined in normal-weight, overweight and obese participants, after taking into account their affect (e.g. depression) and co-existing behaviour (e.g. night-eating, insufficient exercise). Results indicate that body weight (i.e. high BMI) was still linked to aspects of poor sleep quality (i.e. sleep disturbances and low sleep efficiency) even after taking into account the effects of multiple co-existing sleep-disrupting behaviour and affect. The results suggest that overweight/obese people are more likely than normal-weight people to have poor sleep quality but their tendency to engage in sleep-disruptive behaviour (i.e. night-eating) and experience distress may partly explain the relationship between overweight/obesity and impaired sleep.

3. You might use the last sentence to summarise any implications for practice, research, policy, or public health.

You might use the last sentence to summarise any implications for practice, research, policy, or public health.

When examining the relationship between body weight and sleep, researchers should seek to control for the effects of common co-existing behaviour (e.g. night-eating) which might alternately explain the observed relationship between body weight and sleep quality.

## Data Availability

The data that support the findings of this study are available from the corresponding author, [S.E], upon reasonable request.

## Abbreviations

BMI: Body mass index
WHR: Waist to hip ratio
REM: Rapid eye movement
NES: Night-eating syndrome
NEQ: Night-Eating Questionnaire
SSQ: Subjective sleep quality
SOL: Sleep-onset latency
SE: Sleep efficiency
SD: Sleep disturbances
SM: Sleep medication use
DD: Daytime dysfunction
